# Mathematical Modeling of the Effect of Temperature on the Dynamic Characteristics of a Cantilever Beam with Flexible Root

**DOI:** 10.1155/2023/6568120

**Published:** 2023-05-23

**Authors:** Samaher Mohammed Sarhan, Salah Al-Zubaidi

**Affiliations:** ^1^Department of Mechatronics Engineering, Al-Khwarizmi College of Engineering, University of Baghdad, Baghdad 10071, Iraq; ^2^Department of Automated Manufacturing Engineering, Al-Khwarizmi College of Engineering, University of Baghdad, Baghdad 10071, Iraq

## Abstract

This study presents the development of an analytical solution for the dynamic response of a cantilever beam with a flexible root taking into account the influence of temperature. The investigated cantilever beam has a uniform rectangular cross-section with finite lengths. The dynamic response of the cantilever was investigated under three conditions, namely, rigid root, resilient root, and resilient root accompanied by different surrounding temperatures. The selected lengths for the beam were 0.3175, 0.1588, 0.1058, 0.0794, 0.0635, 0.0529, 0.0454, 0.0397, 0.0353, and 0.03175 m. The chosen linear spring coefficients were 0.01, 0.1, 100, and ∞ N/m while rotational spring coefficients were 0.01, 0.1, 100, and ∞ N·m/rad. The surrounding temperatures for the third condition were −100, 25, 100, and 200°C. A MATLAB code was developed to calculate the fundamental natural frequency under different surrounding temperatures and spring coefficients. The proposed mathematical solution was validated with real experimental data and the verification findings revealed a good match between them. For the rigid condition, the finding revealed good matching between the analytical model and experimental results, particularly at the length range of 0.3175−0.1058 m. For the resilient condition, the fundamental natural frequencies were found to be highly affected by decreasing beam length and increased at 100 N/m and 100 N·m/rad and higher coefficients. Finally, there was a reduction in the calculated natural frequencies with increasing temperature.

## 1. Introduction

Mathematical modeling of dynamic behavior for the structural part is an important topic. It is quite necessary to predict the natural frequency during the design stage by deriving a reliable analytical solution. Historically, distinguished efforts have been made in the field of estimation of natural frequencies as well as mode shapes of flexible root cantilever beams [1–6].

Qiao et al. [7] presented an exact solution for solving single and two degrees of freedom for the free flexural vibration of a nonuniform Euler Bernoulli beam. The presented model was efficient in terms of computation with a significant reduction in determinant order compared with other methods. The vibration behavior of a functionally graded beam was investigated by Hein and Feklistova [8] using both the Euler–Bernoulli theory and the Haar technique. Some classic wavelets were applied to simplify and transform the governing equation of the beam system. The beam was investigated under various cross-sections, mass density, rigidity, and different coefficients of translational and rotational stiffness. The Haar wavelet approved its capability through the achieved results where the applied approach was accurate, simple, and soft. Ahmed [9] conducted an experimental and numerical study to analyze the free vibration behavior of a cantilever beam with notched and unnotched geometrical conditions. The beam was made of Kevlar-reinforced epoxy. Good agreement was obtained when the results were compared with published works. The author found a reduction in the computed natural frequencies with increasing notch depth. Also, fiber orientation has some influence on the convergence and divergence between numerical and experimental findings. Majeed et al. [10] modeled a flexible smart cantilever beam based on Euler–Bernoulli and piezoelectric theories using state space and finite element techniques. The aim of the modeled smart structure was to reduce beam vibration and settling time where the achieved results showed effective performance of the proposed technique. Also, a sliding mode observer was designed for vibration suppression of the flexible cantilever beam by Al-Samarraie et al. [11].

Zhang et al. [12] derived an analytical solution for the free vibration analysis of a nonuniform flexible Timoshenko beam with multiple discontinuities. The model results were accurate when verified with the findings of finite element simulation and the literature. A rare case of free vibration for the Timoshenko beam under elastic restraints as stated by Shi et al. [13] was investigated. An exact solution was achieved by applying the Fourier series where simultaneous satisfaction was obtained from the governing equation and boundary condition for any defined level of accuracy. New results for the elastic retrained beam were presented to be used as a benchmark solution for future work. An improved Fourier–Ritz method was adopted by Wang et al. [14] for the free vibration analysis of an axially loaded cantilever beam made of the laminated composite under different boundary conditions. They reported that the model derived using this approach was accurate and reliable and had fast convergence.

Pham and Nguyen [15] employed Euler–Bernoulli's theory for a 3D cantilever beam having a moving hub and flexibility. They applied Hamilton's principles to derive the equation of motion and also used the Galerkin approach to reduce the model order. Both simulation and experimental data validated the derived dynamical model where good matching was achieved. The free vibration problem of the double beam under restrained and coupling conditions was solved by Chen et al. [16] by using an improved Fourier approach. The reliability of the proposed analytical solution was compared with a numerical model. An improved Fourier–Ritz method was applied by Hao et al. [17] to analyze the characteristics of free vibration for the double beam under restrained stiffness and elastic layer conditions. The displacement discontinuities and its derivative were removed using the Fourier series and polynomial function to speed up the model convergence rate. Also, numerical simulation was performed for the investigated beam using different shapes and material properties. The mathematical model was compared with the published works to confirm its reliability and accuracy. Chen and Du [18] took into account the effect of restrained stiffness and rotation speed when deriving an analytical solution based on the Fourier series for a rotational beam. The effectiveness of the analytical solution was approved by comparison with numerical results.

Zhao [19] applied the shape function method to solve the free vibration problem of the axially loaded beams under arbitrary elastic support with concentrated masses and nonconventional boundary conditions. The free vibration governing equation was reformulated by using Dirac's delta function to solve the shape function approach. Good agreement was obtained when the results of the derived mathematical model were with the findings of the published works. Also, Zhao [20] studied the special geometry of double beams having concentrated masses. The author obtained the mode shapes of free and forced vibration for the investigated beam by applying the shape function method. A new orthogonality form condition was derived and the reliability of the model was achieved via comparison with a numerical example.

The free vibration characteristics of the functionally graded material beam were investigated by Kim et al. [21] using the Haar wavelet method. The governing equation was constructed by applying Hamilton's principles to make a generalization of the boundary conditions for the four locations along the beam. Validation of the derived model with published results and numerical findings approves its accuracy and effectiveness.

Paridie et al. [22] developed an artificial neural network (ANN) model to predict the natural frequency of a cantilever beam with various cross-sections and under the effect of magnitude and load location. The ANN model was trained and tested with finite element (FE) simulation data for the beam under investigation. Good matching was achieved between ANN and FE models.

Analytical analysis methods showed and proved their capability by solving various engineering problems. Particularly, the analytical solutions to find the dynamic behavior for vital engineering parts such as cantilever beams have found a wide range of engineering applications (e.g., rotating, blade, cantilever bridges, balconies, cranes, overhanging roofs such as stadium roofs, and shelters). In general, cantilever beams are used in different environments; therefore, providing an analytical solution to find the dynamic response under different environments is a crucial task. A comprehensive search in the available literature was achieved, and it was found that the compound effects of thermal and root stiffness on the dynamic response of the cantilever beam have not been addressed before by any researchers. Therefore, this study investigates the combined effect of temperature and flexible root for a cantilever beam to estimate the dynamic response represented by the fundamental natural frequency. The investigation is conducted based on developing an analytical solution whose results are verified with real findings.

## 2. Development of the Analytical Solution for Free Vibration Cantilever Beam under Surrounding Temperatures and Resilient Conditions

In this section, an analytical model will be developed to identify the fundamental natural frequencies for the cantilever beam, taking into account the effect of a flexible root at one end of the beam and the influence of temperature. The flexible root in this study is taken as linear and rotational springs.

### 2.1. Derivation of the Analytical Solution of the Free Vibration Cantilever Beam

The differential equation that governs the sinusoidal vibrational motion of the cantilever beam is as follows[2]:

(1) $$\begin{array}{c} EI_{xx}Y^{\prime \prime \prime \prime }f_{n}^{4}Y=0\text{,} \end{array}$$

where *E* represents the beam modulus of elasticity, *I*_*xx*_ is the area moment of inertia, and (*f*_*n*_) is the natural frequency.

The prime symbols at *Y* refer to the differentiability per nondimensional parameters; let us call it *Z,* where *Z*=*z*/*L*, where *L* is the total length of the cantilever beam.

Calculating the frequency variable is possible by using the following equation:

(2) $$\begin{array}{c} {f_{n}}^{4}=\frac{\rho A\omega^{2}L^{4}}{E{\left(T\right)\;I}_{xx}}\text{,} \end{array}$$

where *ρ* is the beam density, *A* is the beam cross-sectional area, *ω* is the angular natural frequency, and *T* is the temperature in °C. As stated at the beginning of this section that the cantilever beam under investigation has a resilient root represented by linear and rotational springs at the left end, in this analytical solution, it was taken into consideration the effect of the surrounding temperature, where any change in the surrounding temperature will significantly affect the material properties of the beam (modulus of elasticity). Therefore, it was assumed that the modulus of elasticity of the beam is a function of surrounding temperatures *E*(*T*). This surrounding temperature leads to a change in the magnitude of stiffeness of the structure/beam, and eventually, the values of natural frequency will be changed too. Therefore, this study aims to show how the dynamic response of a cantilever beam is influenced by the change in the surrounding temperature assuming the resilient root conditions (the linear and rotational springs at the root of the beam).

**Figure 1:**
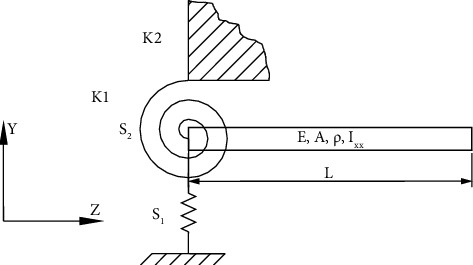
Cantilever beam with the resilient root.

These springs have stiffness of K1 and K2. If boundary conditions are applied to such beam depicted in Figure 1, the following can be attained:

(3) $$\begin{array}{c} At\;z=0\text{,}\\ Y^{\prime \prime \prime }=-S_{T}Y\left(0\right)\text{,}\\ Y^{\prime \prime }\left(0\right)=S_{R}Y^{\prime }\left(0\right)\text{,}\\ At\;z=L\text{,}\\ Y^{\prime \prime \prime }\left(L\right)=0\text{,}\\ Y^{\prime \prime }\left(L\right)=0\text{,} \end{array}$$

where

(4) $$\begin{array}{c} S_{T}=\frac{K1L^{3}}{E\left(T\right)\;I_{xx}}\text{,}\\ S_{R}=\frac{K2L}{E\left(T\right)\;I_{xx}}\text{,} \end{array}$$

where *S*_*T*_ and *S*_*R*_ represent the coefficients of linear and rotational springs, respectively.

The mode shape given in equation (5) satisfies the boundary conditions:

(5) $$\begin{array}{c} y\left(Z\right)=A\left(Z^{4}-4Z^{3}+6Z^{2}+12S_{R}^{*}Z+24S_{T}^{*}\right.\text{.} \end{array}$$

Here,

(6) $$\begin{array}{c} S_{R}^{*}=\frac{1}{S_{R}}\text{,}\\ S_{T}^{*}=\frac{1}{S_{T}}\text{.} \end{array}$$

When we substitute equation (5) into equation (2) while minimizing the resulting error using Galerkin integral, we get the following equation to calculate the fundamental natural frequency:

(7) $$\begin{array}{c} f_{n}^{4}=\frac{36288\;X_{4}}{\left(60840X_{1}+2912X_{2}+72546X_{3}\right)}\text{,} \end{array}$$

where

(8) $$\begin{array}{c} X_{1}=S_{R}^{*2}+12S_{T}^{*2}\text{,} \end{array}$$

(9) $$\begin{array}{c} X_{2}=1+9S_{R}^{*}\text{,} \end{array}$$

(10) $$\begin{array}{c} X_{3}=S_{T}^{*}\left(1+5S_{R}^{*}\right)\text{,} \end{array}$$

(11) $$\begin{array}{c} X_{4}=1+5S_{R}^{*}+20S_{T}^{*}\text{.} \end{array}$$

If the stiffness of linear spring approaches to infinity (*S*_*T*_⟶*∞*), then *S*_*T*_^*∗*^=0 and equation (7) will become

(12) $$\begin{array}{c} f_{n}^{4}=\frac{36288\;\left(1+5S_{R}^{*}\right)}{\left(2912+26208S_{R}^{*}+60840S_{R}^{*2}\right)}\text{.} \end{array}$$

Similarly, if the stiffness of the rotational spring approaches to infinity (*S*_*R*_⟶*∞*), then *S*_*R*_^*∗*^=0 and equation (7) will become

(13) $$\begin{array}{c} f_{n}^{4}=\frac{36288\;\left(1+20S_{T}^{*}\right)}{\left(725760S_{T}^{*}+2912+72576S_{T}^{*2}\right)}\text{.} \end{array}$$

Finally, if the rigid conditions exist (i.e., both *S*_*R*_^*∗*^ and *S*_*T*_^*∗*^ equal zero), the fundamental natural frequency will be

(14) $$\begin{array}{c} f_{n}=1.878854\text{.} \end{array}$$

### 2.2. Development of MATLAB Code

**Figure 2:**
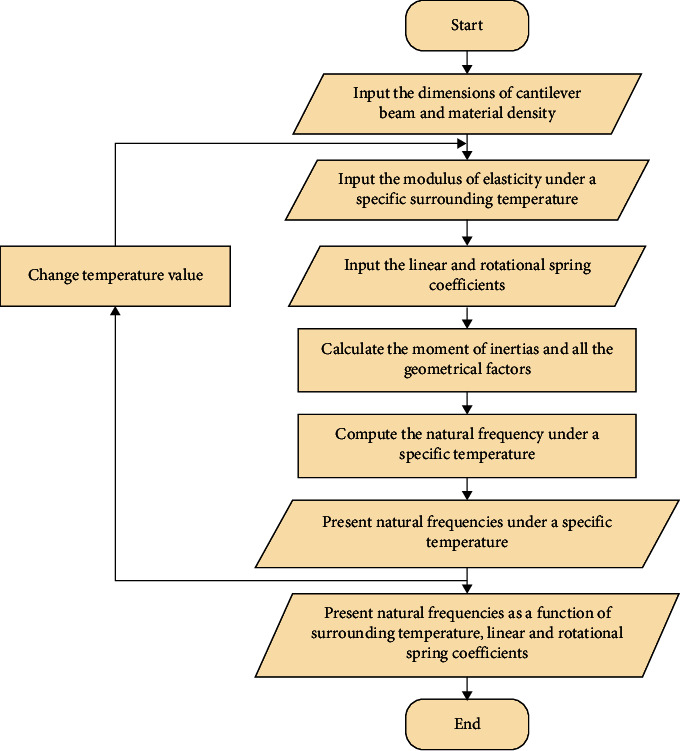
Flowchart of the developed MATLAB code.

To program the differential equation so as to calculate the fundamental natural frequencies of the cantilever beam under the given conditions, a MATLAB code was written to perform this task. The flow chart of the developed code is depicted in Figure 2. It consists of several steps as follows:

1. Enter the dimensions of the cantilever beam and its density
2. Enter the modulus of elasticity for the beam based on the selected surrounding temperatures
3. Enter the linear and rotational stiffness coefficients
4. Determine the moment of inertia and other geometrical parameters
5. Calculate the fundamental natural frequencies using equations (7)–(14) based on the boundary conditions
6. Present the natural frequency under a given surrounding temperature
7. Select another temperature within the specified range and follow steps 2–6
8. Plot the fundamental natural frequencies as a function of linear and torsional stiffness coefficients and temperatures

In fact, the time spent understanding and analyzing the mathematical model of the problem and then formulating the equations of motion, taking into account the thermal and root stiffness (linear and rotational) effects, is considerable (approximately four months). At this stage, it was verified that the accuracy of the results of the analytical solution was verified by comparing it with the results of other researchers who used other approaches. The last stage is to build the code based on the analytical solution to reduce the computational time to minimum. Thus, the execution time (computational cost) was relatively short (approximately 30 min and maybe more according to the case study and the range of the variables) to run and collect the data of the developed MATLAB code.

## 3. Results and Discussion

This section provides and analyzes the achieved findings of the developed analytical solution that was developed essentially to determine the fundamental natural frequency of the cantilever beam. The dynamic response of the cantilever beam was investigated under the following conditions:

1. Cantilever beam with the rigid root
2. Cantilever beam with the resilient root (linear and rotational springs)
3. Cantilever beam with rigid and resilient roots under the effect of temperature.

The results of the abovementioned conditions have been verified with real experimental data as will be seen in the following subsections.

### 3.1. Effect of the Rigid Root on the Dynamic Response of the Cantilever Beam

This subsection presents the results of the calculated fundamental natural frequency for the cantilever beam under free vibration, ambient temperature, and rigid root conditions. The *f*_*n*_ was calculated at different beam lengths, and the results were compared with experimental and theoretical findings [2] Meanwhile, the error % was calculated to show how the results of the derived analytical solution were close or near to the published findings. The three results are tabulated and given in Table 1. On one side, it can be seen that there is a very close matching between the analytical model and the theoretical model. On the other side, there is good matching with the experimental work, particularly at 0.3175−0.1058 m. After that range, some divergence is noticed. The experimental work is normally associated with some noise factor that shifts its results from the theoretical and analytical solutions. It includes human error, environmental conditions, and the accuracy of the utilized apparatuses.

### 3.2. Effect of the Resilient Root on the Dynamic Response of the Cantilever Beam

**Figure 3:**
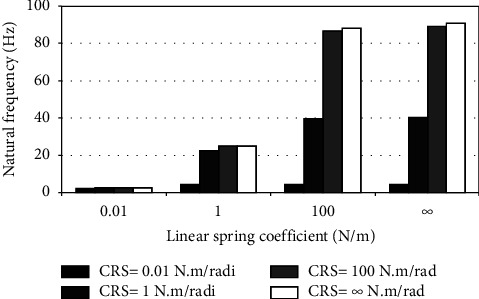
Dynamic response of cantilever beam with the resilient root and different stiffness values (*L* = 0.3175 m).

**Figure 4:**
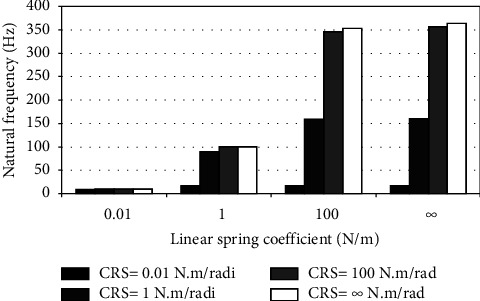
Dynamic response of cantilever beam with the resilient root and different stiffness values (*L* = 0.1588 m).

**Figure 5:**
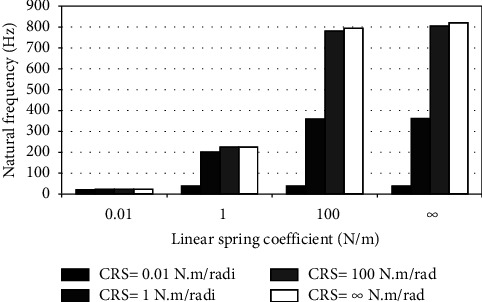
Dynamic response of cantilever beam with the resilient root and different stiffness values (*L* = 0.1058 m).

**Figure 6:**
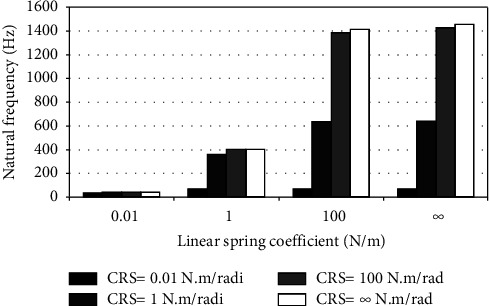
Dynamic response of cantilever beam with the resilient root and different stiffness values (*L* = 0.0794 m).

**Figure 7:**
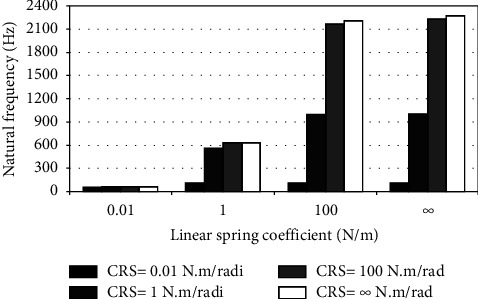
Dynamic response of cantilever beam with the resilient root and different stiffness values (*L* = 0.0635 m).

**Figure 8:**
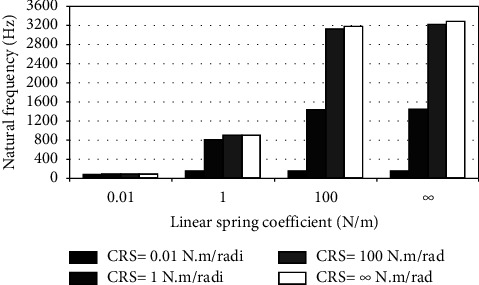
Dynamic response of cantilever beam with the resilient root and different stiffness values (*L* = 0.0529 m).

**Figure 9:**
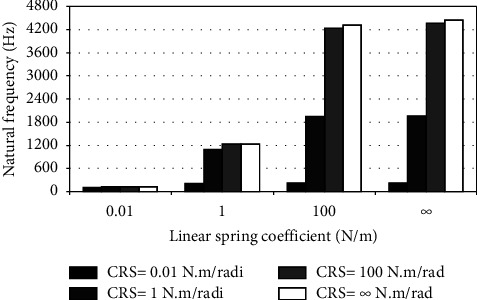
Dynamic response of cantilever beam with the resilient root and different stiffness values (*L* = 0.0454 m).

**Figure 10:**
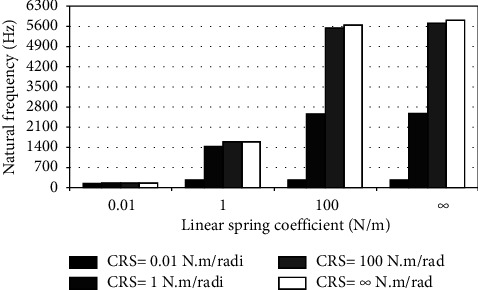
Dynamic response of cantilever beam with the resilient root and different stiffness values (*L* = 0.0397 m).

**Figure 11:**
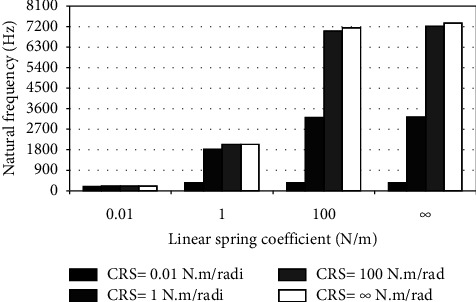
Dynamic response of cantilever beam with the resilient root and different stiffness values (*L* = 0.0353 m).

**Figure 12:**
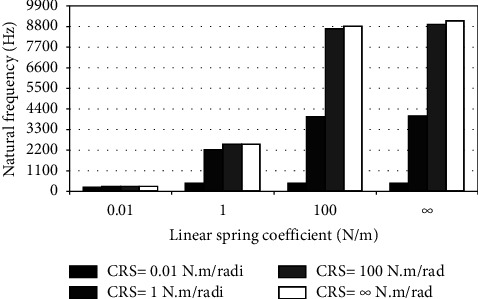
Dynamic response of cantilever beam with the resilient root and different stiffness values (*L* = 0.03175 m).

When the flexible root is attached to one end of the cantilever beam, the dynamic response changes according to the conditions of the impeded resilient root. The effect of both linear and rotational coefficients on the natural frequency is included in the analytical model. The fundamental natural frequencies are plotted against linear and rotational spring coefficients at different beam lengths as depicted in Figures 3–12.

Investigation of Figures 3–12 reveals the following points:

1. The fundamental natural frequency is highly affected by decreasing the cantilever beam length
2. When increasing the linear spring coefficient coactively with the rotational coefficient (CRS), the fundamental natural frequency is highly increased at 100 N/m and 100 N·m/rad and higher coefficients.
3. At a low linear spring coefficient (0.1 N/m), there is no noticeable increase in natural frequency regardless of the values of the rotational spring coefficient and vice versa.

To sum up, there are no high differences in fundamental natural frequencies at a length range of 0.3175 m, regardless of linear and rotational coefficients. Based on the mentioned mathematical formula, it is well known that there is an inverse proportion between frequency and the taken period (time). Therefore, a long cantilever beam takes more time to come back to the original coordinates. In other words, a low natural frequency is produced. Therefore, the length of the cantilever beam must be taken into consideration. Also, to resist the dynamic bending of the beam, a precaution has to be considered in the material selection for the cantilever beam. A more stiff material is preferred to maintain the stability of the beam. On the other hand, at low linear or rotational coefficients (0.01 N/m or 0.01 N·m/rad), the values of natural frequency are low and comparable, and vice versa, for higher linear and rotational coefficients. The higher values of linear and rotational spring coefficients mean that these springs are stiffer than those with low coefficients. Therefore, a cantilever beam with a stiffer spring means that it returns faster to the relaxed position. Quick pull-back motion produces an overshoot, which generates a high amount of oscillation and accordingly a higher natural frequency. In contrast, slowly pulling back to the original position is done by the cantilever beam attached to the springs having low linear and rotational coefficients. Therefore, low resistance to the oscillational motion is obtained because the cantilever beam makes a slow response and hence low natural frequency is produced.

### 3.3. Effect of Temperature on the Dynamic Response of the Cantilever Beam with Resilient Roots

The effect of temperature combined with flexible root is illustrated in this subsection. The fundamental natural frequency was calculated for each temperature-spring coefficient pair. Two alloys were taken as a case study to illustrate the influence of temperature on the fundamental natural frequency, namely, N-based alloy and AA5054 aluminum alloy. The material properties represented by the Young modulus of elasticity were taken as a function of temperature. The selected temperatures were −100, 25, 100, and 200°C, respectively. They include subzero, normal, and high temperatures to show how the dynamic response of the cantilever beam will be impacted under these conditions (i.e., variable temperatures and flexible root).  Table 2 presents the modulus of elasticity (*E*) of those two materials as a function of the selected temperature range.

When *E*-values are replaced in the derived fundamental natural frequency of equation (7), Tables 3 and 4 are obtained. These tables reveal a reduction in fundamental natural frequency with increasing temperature for both materials. As indicated in equation (2), the material properties of the cantilever beam represented by Young's modulus of elasticity were taken as a function of temperature. Consequently, the fundamental natural frequency will be changed accordingly.

## 4. Conclusions and Remarks

In this study, a mathematical model was developed to investigate the effects of linear and rotational spring coefficients conjugated with temperature influence. Based on the presented results and discussion, the following conclusions can be drawn:

1. The analytical model was successfully derived.
2. The proposed analytical solution was verified under rigid conditions and revealed excellent compatibility with the theoretical findings and good matching with experimental results, particularly at the beam length range of 0.3175−0.1058 m.
3. The fundamental natural frequency drastically increased with a decrease in cantilever beam length under rigid conditions.
4. There were no high differences in fundamental natural frequencies for the beam under flexible root conditions.
5. The effect of flexible roots on the dynamic behavior of the cantilever beam started to appear with increasing beam length.
6. The natural frequency was affected by increasing temperature as the beam modulus of elasticity was taken as a function of temperature.

The current study can be extended in future work to find the dynamic response of the rotating cantilever beam (turbomachine blades) working in different environmental conditions with different root stiffness. Also, the influence of the defect in the cantilever beam on the dynamic response under different working conditions can be investigated. Furthermore, the current analytical solution can be enhanced to study the vibration characteristics of a microcantilever working under high-temperature conditions.

## Figures and Tables

**Table 1:** Dynamic response of the cantilever beam with the rigid root.

Length	Current study	Experimental work [2]	Theoretical work [2]	Error %	
				Exp.	Theo.
0.3175	90.67	88.9	91.30	1.99	0.68
0.1588	362.45	345.3	365.10	4.96	0.72
0.1058	816.55	747.8	821.50	9.19	0.60
0.0794	1451.64	1300.0	1460.50	11.66	0.60
0.0635	2268.35	1968.3	2282.20	15.44	0.60
0.0529	3268.49	2736.8	3286.30	19.42	0.54
0.0454	4437.58	3594.7	4473.10	23.44	0.79
0.0397	5803.33	4550.0	5842.40	27.54	0.66
0.0353	7340.22	5513.5	7394.30	33.13	0.73
0.03175	9073.7	6731.80	9128.70	34.78	0.60
Min	90.67	8.9	91.3	1.99	0.79
Max	9073.7	6731.8	9128.7	34.78	0.54

**Table 2:** Young modulus of elasticity for *N*-based alloy and AA5054 aluminum alloy for different temperatures.

No.	Alloy	Young modulus of elasticity for different temperatures			
		−100	25	100	200
1	Ni-Fe-Cr 800	207	196	193	186
2	AA 5054	74.1	70.3	67	55.7

**Table 3:** Fundamental natural frequency of Ni-Fe-Cr 800 alloy as a function of temperature and flexible root.

		Temperature (°C)			
		−100	25	100	200
Rotational spring coefficient (N·m/rad)	0.01	458.1	446.23	442.823	434.793
	1.0	4020.85	3990.3	3917.95	3844.23
	100.0	8828.13	8670.8	8507.73	8270.08
	∞	9003.64	8837.3	8590.50	8580.50

Linear spring coefficient (100 N/m).

**Table 4:** Fundamental natural frequency of AA 5054 alloy as a function of temperature and flexible root.

		Temperature (°C)			
		−100	25	100	200
Rotational spring coefficient (N·m/rad)	0.01	11.9029	11.5937	11.3183	10.3198
	1.0	106.4389	103.6738	101.2112	92.28239
	100.0	231.2885	225.28	219.9288	200.5268
	∞	235.7298	229.6059	224.152	204.3774

Linear spring coefficient (100 N/m).

## Data Availability

The data that support the findings of this study are available from the corresponding author upon reasonable request.
